# The bidirectional relationship between periodontal disease and pregnancy *via* the interaction of oral microorganisms, hormone and immune response

**DOI:** 10.3389/fmicb.2023.1070917

**Published:** 2023-01-26

**Authors:** Xingyue Wen, Xiangqing Fu, Chongjun Zhao, Lei Yang, Ruijie Huang

**Affiliations:** State Key Laboratory of Oral Diseases, National Clinical Research Center for Oral Diseases, Department of Pediatric Dentistry, West China Hospital of Stomatology, Sichuan University, Chengdu, China

**Keywords:** periodontal diseases, periodontal pathogens, adverse pregnancy, inflammation, immune response

## Abstract

Periodontal disease has been suggested to be linked to adverse pregnancy outcomes such as preterm birth, low birth weight, and preeclampsia. Adverse pregnancy outcomes are a significant public health issue with important clinical and societal repercussions. This article systematically reviews the available epidemiological studies involving the relationship between periodontal disease and adverse pregnancy outcomes over the past 15 years, and finds a weak but independent association between adverse pregnancy outcomes and periodontal disease. The bidirectional association and the potential mechanisms are then explored, focusing on three possible mechanisms: inflammatory reaction, oral microorganisms and immune response. Specifically, elevated systemic inflammation and increased periodontal pathogens with their toxic products, along with a relatively suppressed immune system may lead to the disruption of homeostasis within fetal-placental unit and thus induce adverse pregnancy outcomes. This review also explains the possible mechanisms around why women are more susceptible to periodontal disease. In conclusion, pregnant women are more likely to develop periodontal disease due to hormonal changes, and periodontal disease has also been suspected to increase the incidence of adverse pregnancy outcomes. Therefore, in order to lessen the risk of adverse pregnancy outcomes, both obstetricians and dentists should pay attention to the development of periodontal diseases among women during pregnancy.

## 1. Introduction

In 2013, the Joint EFP/AAP (European Federation of Periodontology/American Academy of Periodontology) Workshop published an updated consensus report focusing on periodontal diseases and adverse pregnancy outcomes (Sanz et al., 2013).

Adverse pregnancy outcomes are serious public health issues with wide-ranging social and economic effects (Bobetsis et al., 2020), and many studies have shown their association with periodontal diseases. Preterm birth (PT), which is defined as delivery prior to 37 full weeks (<259 days), is the primary cause of neonatal death in the first 4 weeks of life (Zi et al., 2014; Iheozor-Ejiofor et al., 2017). Low birth weight (LBW), defined as a weight less than 2,500 g at birth, is typical for infants born preterm and/or with intrauterine restricted growth conditions (IUGR). Additionally, LBW infants are more likely to experience adverse outcomes, such as an increased mortality rate. Preeclampsia (PE) is a multisystem pregnancy condition that affects around 2–10% of pregnant mothers and is a major risk factor for preterm birth and slow infant growth. Hypertension and proteinuria in pregnant women were characterized after the 20th week of gestation (Zi et al., 2014; Gare et al., 2021). It should be noted that, “adverse pregnancy outcome” is a broad term that extends beyond these noted conditions.

Periodontal disease was identified as a possible risk factor for PT as early as 1996 (Offenbacher et al., 1996). Particularly since Han et al. (2006) reported direct evidence of the first oral- utero translocation in 2006, suggesting that the oral cavity was the source of the *Bergeyella* strain found in the patient’s intrauterine illness, a great deal of effort has been placed in the field of association between oral health and pregnancy over the last 16 years. While some facets of the association between periodontal disease and adverse pregnancy outcomes have been clarified, the potential relationship between them remains controversial and the underlying mechanisms must be better revealed and elucidated.

Given the relatively high incidence of worsened dental health among pregnant women and the devastating consequences of adverse pregnancy outcomes, combined with the reality that oral diseases are largely both curable and avoidable, this review will focus on recent literature reporting the relationship between oral health and pregnancy complications. It will also discuss possible mechanisms of this association. This review assesses the issue from a bidirectional and reciprocal relationship; that is, not only how oral health affects the outcomes of pregnancy, but also how some of the physiological changes that occur during pregnancy can alter oral cavity conditions.

## 2. Deterioration of periodontal status correlates with adverse pregnancy outcomes

### 2.1. Epidemiological studies support

**Figure 1:**
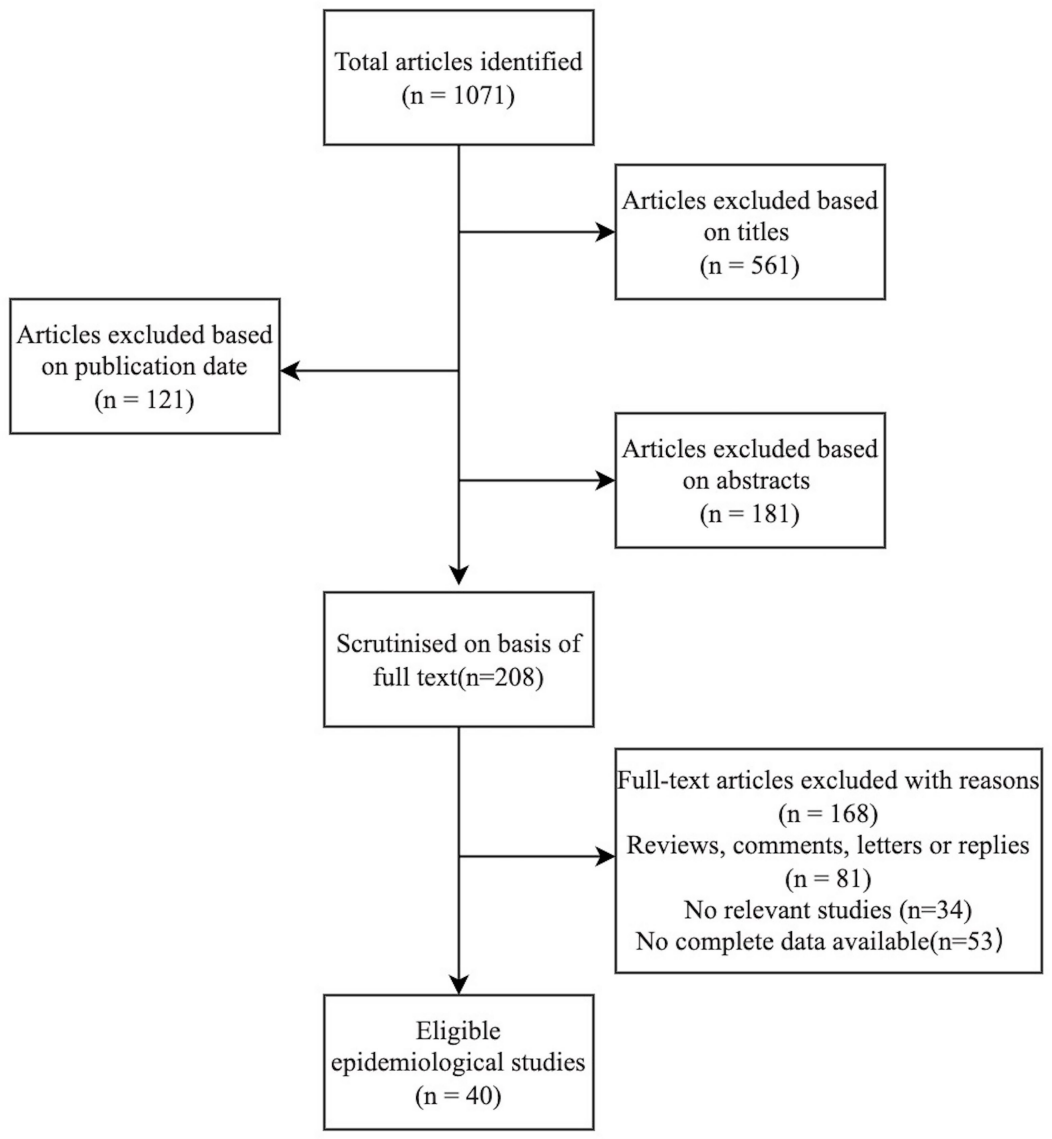
Flowchart of search process and reasons for exclusion.

According to epidemiological evidence, preterm birth, low birth weight, pre-eclampsia, and other adverse pregnancy outcomes may be associated with periodontal disease. Forty studies published in the last 15 years and indicating a relationship between periodontal disease and adverse pregnancy outcomes were identified after a search on PubMed following the process shown in the flowchart (Figure 1) below. These studies have been summarized in Table 1. Inclusion criteria were as follows: (1) original publications reporting data from randomized and non-randomized controlled trials, case–control, cross-sectional or cohort studies on the association between periodontal condition and adverse pregnancy outcomes; (2) women during reproductive age; (3) sufficient data such as relative risk (RR), the odds ratio (OR), hazard ratio (HR), *p* values or 95% confidence interval (CI) were available (4) choose the most recent and comprehensive study when there are overlapping ones. Exclusion criteria were as follows: (1) inadequate or confusing case definitions and unavailable data; (2) papers with abstract only; (3) animal research; (4) literature reviews, comments, letters or replies; (5) languages other than English.

**Table 1:** Epidemiological studies that reported the association between adverse pregnancy outcomes and periodontal disease published in the last 15 years.

Reference	Sample Size	Sample analyses	Main results
Siqueira et al. (2007)	IUGR *n* = 77 LBW *n* = 235 PT *n* = 238 Control *n* = 1,042	Maternal periodontitis was retained in the final model for PT [odds ratio (OR) = 1.77; 95% confidence interval (CI): 1.12–2.59], LBW (OR = 1.67; 95% CI: 1.11–2.51), and IUGR (OR = 2.06; 95% CI: 1.07–4.19) after adjusting for variables of interest.	Periodontal disease is linked to an increased risk for PT, LBW, and IUGR.
Bassani et al. (2007)	Cases *n* = 304 Controls *n* = 611	Odds ratio were 0.93 [95% confidence interval (CI): 0.63–1.41] for LBW and 0.92 (95% CI: 0.54–1.57) for pre-term LBW in the presence of periodontitis, after adjustment for maternal age, previous pregnancies, pre-natal care, smoking, previous low birth or premature birth and other medical conditions.	The findings refute the theory that periodontal disease and IUGR, LBW, and PT are related.
Le et al. (2007)	Cases *n* = 130 Controls *n* = 260	Periodontitis was significantly associated with PT (adjusted OR = 4.47, 95% CI: 2.43–8.20).	Periodontitis may increase the risk of PT.
Marakoglu et al. (2008)	*n* = 48	Periodontitis (OR: 3.6, 95% CI: 1.06–12.18) together with bacterial vaginosis (OR: 11.57, 95% CI: 1.26–105.7) were independent risk factors of a preterm low birth weight.	A poor periodontal health status of the mother may be a potential risk factor for a preterm low birth weight.
Saddki et al. (2008)	*n* = 427	The relative risk of having LBW infants was 4.27 times higher for women with periodontitis compared with those without periodontitis (95% CI: 2.01–9.04). After adjustment for potential confounders using multiple logistic regression analysis, significant association was found between maternal periodontitis and LBW (OR = 3.84; 95% CI: 1.34–11.05).	Pregnant women with periodontitis are at a significantly higher risk of delivering LBW infants.
Srinivas et al. (2009)	*n* = 786	There was no association between periodontal disease and the composite outcome [adjusted odds ratio (AOR), 0.81; 95% confidence interval (CI), 0.58–1.15; *p* = 0.24], preeclampsia (AOR = 0.71; 95% CI: 0.37–1.36; *p* = 0.30), or preterm birth (AOR = 0.77; 95% CI: 0.49–1.21; *p* = 0.25) after adjusting for relevant confounders.	This large prospective study failed to demonstrate an association between periodontal disease and adverse pregnancy outcomes.
Cruz et al. (2009)	LBW *n* = 164 Controls *n* = 384	A statistically significant association was found between periodontal disease and LBW (unadjusted OR = 1.74; 95% CI: 1.19–2.54).	The findings suggest an association between periodontal disease and LBW.
Vogt et al. (2010)	*n* = 327	Periodontal disease was linked to a higher risk of PT (RRadj. 3.47, 95% CI: 1.62–7.43) and LBW (RRadj. 2.93, 95% CI: 1.36–6.34).	Periodontal disease was a risk factor for PT and LBW among Brazilian low risk pregnant women.
Guimarães et al. (2010)	*n* = 1,207	Periodontal disease was associated with PT (*n* = 161; mild–moderate and extreme) or extreme PT (*n* = 15) by ordinal logistic regression [definition 1: odds ratio (OR) = 1.83, 95% confidence interval (CI): 1.28–2.62; definition 2: OR = 2.37, 95% CI: 1.62–3.46].	Periodontal disease is associated with a premature or extremely premature birth.
Rakoto-Alson et al. (2010)	*n* = 204	The rates of periodontitis were considerably higher in PT (78.6%, *p* < 0.001) and LBW (77.3%, *p* < 0.001) groups than in the full-term (8.6%), normal weight (16.5%), and normal birth (2.7%) groups.	Periodontitis was significantly associated with PT and LBW.
Shetty et al. (2010)	*n* = 130	Multiple logistic regression demonstrated that periodontitis both at enrollment (OR = 5.78, 95% CI: 2.41–13.89) as well as within 48 h of delivery (OR = 20.15, 95% CI: 4.55–89.29), may be associated with an increased risk of preeclampsia.	There was significant difference between the PE and normotensive groups in the distribution of periodontitis.
Baskaradoss et al. (2011)	Cases *n* = 100 Controls *n* = 200	Logistic regression analysis indicated a risk of nearly threefold for PT in mothers with periodontitis [adjusted odds ratio (OR(a)) = 2.72; 95% confidence interval (CI): 1.68–6.84].	Periodontal disease is a possible risk factor for PT in this population.
Ali and Abidin (2012)	*n* = 73	Of those with periodontal disease, 4 (10.8%) had PT delivery and 3 (8.1%) had LBW infants. None of the PD variable means or PD status associated significantly with either of the two groups (*p* > 0.05).	Periodontal disease was not shown to be a risk factor for PT or LBW infant.
Guimarães et al. (2012)	*n* = 1,206	Ordinal logistic regression showed that maternal periodontitis was associated with LBW and VLBW [odds ratio (OR) = 2.0; 95% confidence interval (CI) 1.39–2.90, when considering periodontitis definition 2]; and with LBW (OR = 1.65; 95% CI: 1.15–2.36, when considering periodontitis definition 1).	Maternal periodontitis was associated with LBW, as well as with VLBW.
Moura da Silva et al. (2012)	*n* = 574	The multivariate logistic regression analysis showed that, after adjustment for other risk factors, periodontitis remained an independent risk factor for PE [adjusted odds ratio (OR) = 8.60, confidence interval (CI) = 3.92–18.88, *p* < 0.001 and adjusted OR = 2.03, 95% CI = 1.43–2.90, *p* < 0.001].	Periodontitis was a PE risk factor in the population that was the subject of the study.
Taghzouti et al. (2012)	PE *n* = 92 Controls *n* = 245	After adjusting for confounding variables, periodontitis remained not associated with PE (adjusted OR = 1.13, 95% CI = 0.59–2.17).	This study does not support the hypothesis of an association between periodontal disease and PE.
Alchalabi et al. (2013)	*n* = 277	Women with periodontal disease were at higher risk for developing PE, PT, and LBW. The rate of PE in women with periodontal disease was 18.6 percent compared to 7.3 percent in the control group (*p* = 0.005) (OR = 2.7, 95% CI: 1.2, 6.0). The OR for PB was (4.4, 95% CI: 1.7–11.7) and for LBW was (3.5, 95% CI: 1.6–7.5).	Periodontal disease is associated with increased risk of PE, PT, and LBW in healthy Jordanian women.
Kumar et al. (2013)	*n* = 340	Periodontitis was found to be significantly associated with PE, IUGR, PT and LBW with odds ratios (95% confidence interval) of 7.48 (2.72–22.42), 3.35 (1.20–9.55), 2.72 (1.30–5.68), and 3.03 (1.53–5.97), respectively.	An increased risk of PE, IUGR, PT, and LBW is linked to maternal periodontitis.
Haerian-Ardakani et al. (2013)	LBW *n* = 44 Controls *n* = 44	Among the known risk factors of LBW babies, history of previous LBW infant among case mothers reached statistical significance (*p* = 0.0081). Mothers of LBW infants had less healthy areas of gingiva (*p* = 0.042), and more deep pockets (*p* = 0.0006).	Periodontal disease was associated with LBW.
Santa Cruz et al. (2013)	*n* = 170	One hundred and seventy women were included in the study (116 non-periodontitis and 54 with periodontitis). The incidence of preterm (PT) and low-birth weight (LBW) was 2.94 and 3.53%, respectively. Periodontal status did not show any association with adverse pregnancy outcomes.	The clinical periodontal condition was not associated with adverse pregnancy outcomes in a Spanish Caucasian population with medium-high educational level.
Kothiwale et al. (2014)	*n* = 770	The univariate logistic regression analysis indicated that mothers with a probing pocket depth (PPD) > 6 mm (OR = 2.21, 95% CI [1.07–4.55], *p* = 0.032) had a higher risk of giving birth to low birth weight infants.	Periodontitis significantly influenced LBW. An increase in the incidence of PT was linked to a worsening of periodontal disease.
Varshney and Gautam (2014)	PE *n* = 20 Controls *n* = 20	PE cases were more likely to develop periodontal disease (*p* < 0.05). 30% of the test group and 65% of the case group had periodontal disease (*p* < 0.05) which had shown that pre-eclamptic cases were 4.33 times more likely to have periodontal disease (odds ratio = 4.33).	Maternal oral status was determined to be associated with an increased risk of PE.
Ha et al. (2014)	*n* = 283	After adjusting for all confounders, the adjusted odds ratio of periodontitis for PE was 5.56 (95% confidence interval of 1.49–20.71).	There was a significant relationship between periodontitis and the occurrence of PE among never-smokers.
Bulut et al. (2014)	Cases *n* = 50 Controls *n* = 50	There were no statistically significant differences between the cases and controls with regard to periodontal disease and PT (OR = 1.48; 95% CI = 0.54–4.06).	Periodontitis was not a possible risk factor for PT.
Jacob and Nath (2014)	LBW *n* = 170 Controls *n* = 170	The multivariate logistic regression model demonstrated that periodontal disease is a significant independent risk factor with an adjusted odds ratio (aOR) of 2.85 for the LBW group [95% confidence interval (CI): 1.62–5.5].	Periodontitis represents a strong, independent, and clinically significant risk factor for LBW.
Basha et al. (2015)	*n* = 340	Logistic regression analysis showed a strong association between periodontitis and poor pregnancy outcomes after adjusting for all variables with OR = 4.54 (95% CI = 1.98–5.46) for PT, and 5.32 (95% CI = 2.01–6.79) for LBW.	Periodontitis is an independent risk factor for poor pregnancy outcome.
Tellapragada et al. (2016)	*n* = 726	Rates of PT and LBW in the study population were 7.6 and 11.4%, respectively. LBW and maternal periodontitis: RR, 3.38 (95% CI: 1.6–6.9; *p* = 0.003) PT and maternal periodontitis: RR, 2.39 (95% CI: 1.1–4.9; *p* = 0.002) PT, LBW and maternal periodontitis: RR, 3.29 (95% CI: 1.8–5.7; *p* < 0.001)	The study findings underscore the need to consider screening for periodontal infections during routine antenatal care in developing countries.
Khan et al. (2016)	LBW *n* = 80 Controls *n* = 80	On multivariate logistic regression, periodontitis was found to be a significant independent risk factor for LBW (aOR: 3.173, 95% CI: 1.429–7.047, *p* = 0.005).	Periodontal disease is associated with LBW.
Soucy-Giguère et al. (2016)	*n* = 273	Women with periodontal disease were more likely to develop PE, and this association remained significant after adjustment for potential confounders (adjusted RR 5.89; 95% CI: 1.24–28.05).	Periodontal disease is associated with PE.
Souza et al. (2016)	LBW *n* = 269 Controls *n* = 682	Periodontitis did not show an association with LBW (ORcrude = 0.92; 95% CI: 0.63–1.35), even after adjustment for the following confounders.	The findings of this study showed no association between maternal periodontal disease and LBW.
Govindasamy et al. (2017)	*n* = 3,500	On comparison between the case and control groups, none of periodontal parameters showed significant association except for the crude association observed in Group-4 for mild periodontitis (OR = −1.561; *p* = 0.000) and PT/LBW.	Periodontitis is not a significant independent risk factor for PT and/or LBW.
Lohana et al. (2017)	*n* = 300	LBW and maternal periodontitis: there was a statistical association between the level of periodontal disease severity and LBW (*p* < 0.001).	Periodontal disease is a potential risk factor for preterm low birth weight babies of pregnant women.
Figueiredo et al. (2019)	*n* = 142	For neonates, the chance of IUGR was 11.53 times higher for pregnant women with periodontal disease (OR = 11.53, *p* = 0.041).	The periodontal disease increased the chance of IUGR.
Kinane et al. (2020)	*n* = 1,117	Periodontal disease was significantly associated with higher odds of pre-eclampsia [adjusted Odds Ratio 95 percent Confidence Interval (aOR = 4.12; 95% CI: 2.20–7.90)], LBW (aOR = 2.41; 95% CI: 1.34–4.33) and PT (aOR = 2.32; 95% CI: 1.33–4.27). There was no significant association between periodontal disease and preterm premature rupture of membranes (aORs 1.83; 95% CI: 0.75–4.21) and PE (3.71; 95% CI: 0.80–17.13).	Maternal periodontal disease is a potential independent risk indicator for PE, LBW, and PT.
Caneiro et al. (2020)	*n* = 158	The duration of pregnancy in healthy patients was 38.78 ± 4.49 weeks, and in patients with periodontitis 37.81 ± 4.89 weeks, with no statistical difference (*p* > 0.05).	Periodontitis was not associated with PT in a Spanish Caucasian cohort.
Erchick et al. (2020)	PT *n* = 197 Controls *n* = 1,197	In the adjusted regression model, increasing extent of gingival inflammation was associated with a non-significant increase in risk of PT (BOP ≥30 percent vs. no BOP: adjusted relative risk (aRR) 1.37, 95% CI: 0.81–2.32).	Periodontal disease were risk factors for PT.
Oliveira et al. (2021)	*n* = 2,474	Periodontitis was associated with a risk almost two times higher of PT compared with healthy pregnant women (RR = 1.93; 95% CI: 1.09–3.43).	Periodontal disease increased the risk of PT.
Shaggag et al. (2022)	PT *n* = 165 Control *n* = 165	Women who had periodontitis had double the odds of having PT compared to women who had no periodontitis (adjusted Odd Ratio = 2.05, 95% Confidence Interval = 1.20–3.52).	The association between periodontitis and PT was significant.
Shah et al. (2022)	*n* = 200	Data was analyzed using SPSS. Low birth weight preterm birth was associated with education level and family size (*p* < 0.05). There was no association between maternal chronic apical periodontitis and low birth weight preterm birth (*p* > 0.05).	There was no association between maternal chronic apical periodontitis and low birth weight preterm birth.
Lee et al. (2022)	*n* = 1,757,774	After variables adjustment, the advanced periodontal disease group had OR of 1.09 (95% CI: 1.07–1.11) for PT, the mild periodontal disease group had OR of 1.05 (95% CI: 1.04–1.06), while no-periodontal disease group had OR of 1.	Increased periodontal disease severity was related to higher risk of PT.

IUGR, intrauterine growth restriction; LBW, low birth weight (<2,500 g); VLBW, very low birth weight (<1,500 g); PE, preeclampsia; PT, preterm birth (gestational age < 37 weeks).

A total of 1,071 articles were identified in the first search. After headlines, abstract, and data screening, 863 articles were excluded for being irrelevant. The remaining 208 papers were read and assessed in their entirety, and 40 articles (*n* = 1,781,311 participants) were selected for analysis. These identified studies focused on low birth weight, preterm birth, preeclampsia and intrauterine growth restriction. Of the selected studies, 31 suggested a correlation between adverse pregnancy outcomes and periodontal disease (ORs ranging from 0.92 to 20.15) and nine found no evidence of an association (ORs ranging from 0.71 to 1.56). A summary of evidence has been listed in Table 2.

**Table 2:** A summary of evidence about periodontal disease and adverse pregnancy outcomes.

Outcomes	Studies show “positive” effect		Studies show “no” effect	
	No.	Studies	No.	Studies
PT	17	6 case–control studies (Le et al., 2007; Siqueira et al., 2007; Marakoglu et al., 2008; Baskaradoss et al., 2011; Kothiwale et al., 2014; Shaggag et al., 2022); 2 cross-sectional studies (Guimarães et al., 2012; Kinane et al., 2020); 9 cohort studies (Rakoto-Alson et al., 2010; Vogt et al., 2010; Alchalabi et al., 2013; Kumar et al., 2013; Basha et al., 2015; Tellapragada et al., 2016; Erchick et al., 2020; Oliveira et al., 2021; Lee et al., 2022).	8	3 case–control studies (Bassani et al., 2007; Bulut et al., 2014; Shah et al., 2022); 1 cross-sectional study (Govindasamy et al., 2017); 4 cohort studies (Srinivas et al., 2009; Ali and Abidin, 2012; Santa Cruz et al., 2013; Caneiro et al., 2020).
LBW	17	7 case–control studies(Siqueira et al., 2007; Marakoglu et al., 2008; Cruz et al., 2009; Haerian-Ardakani et al., 2013; Jacob and Nath, 2014; Khan et al., 2016; Souza et al., 2016); 2 cross-sectional studies (Guimarães et al., 2012; Kinane et al., 2020); 7 cohort studies (Rakoto-Alson et al., 2010; Vogt et al., 2010; Alchalabi et al., 2013; Kumar et al., 2013; Basha et al., 2015; Tellapragada et al., 2016; Lohana et al., 2017); 1 randomized controlled trial (Saddki et al., 2008).	5	2 case–control studies (Bassani et al., 2007; Shah et al., 2022); 1 cross-sectional study (Govindasamy et al., 2017); 2 cohort studies (Ali and Abidin, 2012; Santa Cruz et al., 2013).
PE	8	3 case–control studies (Shetty et al., 2010; Moura da Silva et al., 2012; Varshney and Gautam, 2014); 1 cross-sectional studies (Kinane et al., 2020); 4 cohort studies (Alchalabi et al., 2013; Kumar et al., 2013; Varshney and Gautam, 2014; Soucy-Giguère et al., 2016).	2	1 case–control study (Taghzouti et al., 2012); 1 cohort study (Srinivas et al., 2009).
IUGR	3	1 case–control studies (Siqueira et al., 2007); 2 cohort studies (Kumar et al., 2013; Costa et al., 2019).	1	1 case–control study (Bassani et al., 2007).

Adverse pregnancy outcomes include PT, LBW/VLBW, PE, IUGR, spontaneous miscarriage, gestational diabetes, fetal injury and stillbirth. For PT, one cohort study (including 1,757,774 pregnant women) by Lee et al. (2022) showed that the more severe the periodontal disease, the higher the risk of PT after variables adjustment. Another cohort study conducted in Africa indicated that women with periodontal disease were twice as likely to PT as women with healthy oral conditions. Low hemoglobin was also demonstrated to be a risk factor for PT (Shaggag et al., 2022). For LBW, Kinane et al. (2020) recruited 1,117 women with singleton delivery and found that periodontal disease was an independent risk indicator for LBW (aOR = 2.41; 95% CI: 1.34–4.33 after adjustment for age, parity, and previous history). However, a prospective cross-sectional study including 3,500 pregnant women suggested no significant association between periodontal parameters and LBW (Govindasamy et al., 2017). For PE, Varshney and Gautam (2014) found that women with PE are 4.33 times more likely to have periodontal disease than normal pregnancies. Coincidentally, the prospective study by Ha et al. (2014) to evaluate the link between periodontal health and PE in a never-smoking population showed that there was a significant relationship between periodontal disease and PE among pregnant women at 21–24 weeks of gestation. Maternal periodontal disease and IUGR have rarely been investigated in recent studies, but a retrospective cohort study has shown an 11.53 times increase in the probability of IUGR in pregnant women with severe periodontal disease (Figueiredo et al., 2019). In addition, adverse pregnancy outcomes such as spontaneous miscarriage, gestational diabetes, and stillbirth have been studied, but their association with periodontal disease requires further investigation (Bobetsis et al., 2020). The evidence suggests there is a correlation between periodontal disease and adverse pregnancy outcomes. Although 31 articles indicated significant association between adverse pregnancy outcomes and periodontal disease, other conflicting studies did not report results of statistical significance between them, possibly due to variations in clinical parameters of periodontal disease assessment, variability in study populations, inclusion of pregnant women in different gestation periods, variation in disease severity and extension, inadequate data analyses, discrepancies of types of the diseases (aggressive and chronic periodontal disease) and so on (Zi et al., 2014; Figuero et al., 2020). Overall, much of the research points to a link between worsening periodontal health and a higher rate of adverse pregnancy outcomes (Bobetsis et al., 2020).

Periodontal conditions can affect pregnancy outcomes. What exactly are the biological mechanisms behind this? Two major pathways have been hypothesized in the consensus report from the Joint EFP/AAP Workshop on periodontitis and systematic diseases. One is indirect mechanisms, largely mediated by periodontitis-associated elevation of inflammatory mediators which can break the homeostasis of placental barrier. The other is direct mechanisms, mainly associated with oral microorganism translocation and the toxic component secretion on site. Both can trigger a metastatic infection within the fetal-placental unit (Figuero et al., 2020). In addition, based on the literature review, an aberrant shift in maternal immune response during pregnancy also seems to play a role in adverse pregnancy outcomes (Zi et al., 2014). These three postulated pathways will be described below.

### 2.2. Indirect infection: Elevated systemic inflammation by periodontal pathogens impacts the fetal-placental unit

A series of studies have been conducted to explore the association between elevated serum levels of inflammatory cytokines and adverse pregnancy outcomes. According to some studies, women with subclinical intra-amniotic infection had considerably higher levels of maternal blood inflammation-associated cytokines than healthy women. For example, a study by Perunovic et al. (2016) showed that PT women had worse periodontal parameters and significantly increased levels of prostaglandin E2 (PGE2) and interleukin-6 (IL-6), both of which are labor triggers and therefore contribute to the preterm birth. However, few contradicting findings without discernible differences have also been published. An observational case/control study by Mesa et al. (2016) showed that no relationship was found between PT/LBW and the markers of systemic inflammatory response assessed such as tumor necrosis factor-α (TNF-α) and interleukin-1β (IL-1β).

In the subgingival region, gram-negative microaerophilic and anaerobic bacteria generate large quantities of proinflammatory mediators. Increased production of inflammatory mediators of periodontal origin may initiate metastatic inflammation including the placenta-fetal unit by blood circulation (Zi et al., 2014; Figuero et al., 2020). Studies in animals showed that periodontal pathogen infections in mothers raise levels of circulating IL-1β, IL-6, interleukin-17 (IL-17), and TNF-α and cause PT (Ao et al., 2015). Periodontal infections and their byproducts lead to the release of Cyclooxygenase-2 (COX-2), IL-8, interferon-γ (IFN-γ), and TNF-α secretion and/or apoptosis in placental tissues/cells *in vitro* models (Ren and Du, 2017). Focusing on a few specific inflammation-related factors, TNF-α has been identified as a potential mediator of overexpression of endothelial activation and injury, a key pathogenetic mechanism of PE (Fitzmaurice et al., 2004). IL-6 may be associated with polycystic ovary syndrome, which increases the risk of PE and PT (Piltonen, 2016). Elevated levels of proinflammatory cytokines, in particular interleukins IL-6, IL-1β, and TNF-α are associated with PT as compared to levels found at term birth (Lyon et al., 2010). In addition, elevated levels of prostaglandins in the chorion can lead to cervical ripening and uterine contractions, ultimately leading to an increased risk of PT (Bobetsis et al., 2020).

As mentioned above, increased periodontal origin inflammatory mediators may initiate metastatic inflammation in the placenta. Additionally, the periodontal microorganisms together with their byproducts can trigger an inflammatory cascade *via* hematogenous dissemination. Microbial pattern recognition receptors, such as Toll-like Receptors (TLR), recognize these circulating microbes and signal pro-inflammatory pathways in the placenta (chorion, metamorphosis and trophoblast cells; Guleria and Pollard, 2000). Furthermore, signaling the periodontal pathogens in gingival tissues helps release additional inflammatory agents that can spread through the system. The final result is that circulating microorganisms together with their by-products initiate an inflammatory response at the fetal-placental unit, indirectly (Bobetsis et al., 2020; Figuero et al., 2020; Genco and Sanz, 2020).

Regardless of how inflammatory response is induced, the exacerbation of inflammatory processes causes the shift of the uterus from a quiescent to a contractile state, which may result in PT, fetal injury, LBW and so on (Romero et al., 2014; Zi et al., 2014).

Notably, bacteria and proinflammatory cytokines in the infected periodontal tissues are released into the systemic circulation and can increase C-reactive protein levels through an acute response in the liver of pregnant women, which can lead to adverse pregnancy outcomes such as PE, PT and IUGR (Paraskevas et al., 2008; Bobetsis et al., 2020). Acute-phase reactants can trigger secondary reactions, known as metastatic inflammation, in the fetal-placental unit. In other words, intrauterine inflammatory response can be amplified by increased plasma C-reactive protein, which can lead to adverse pregnancy outcomes through tissue damage, complement activation and induction of proinflammatory cytokines.

**Figure 2:**
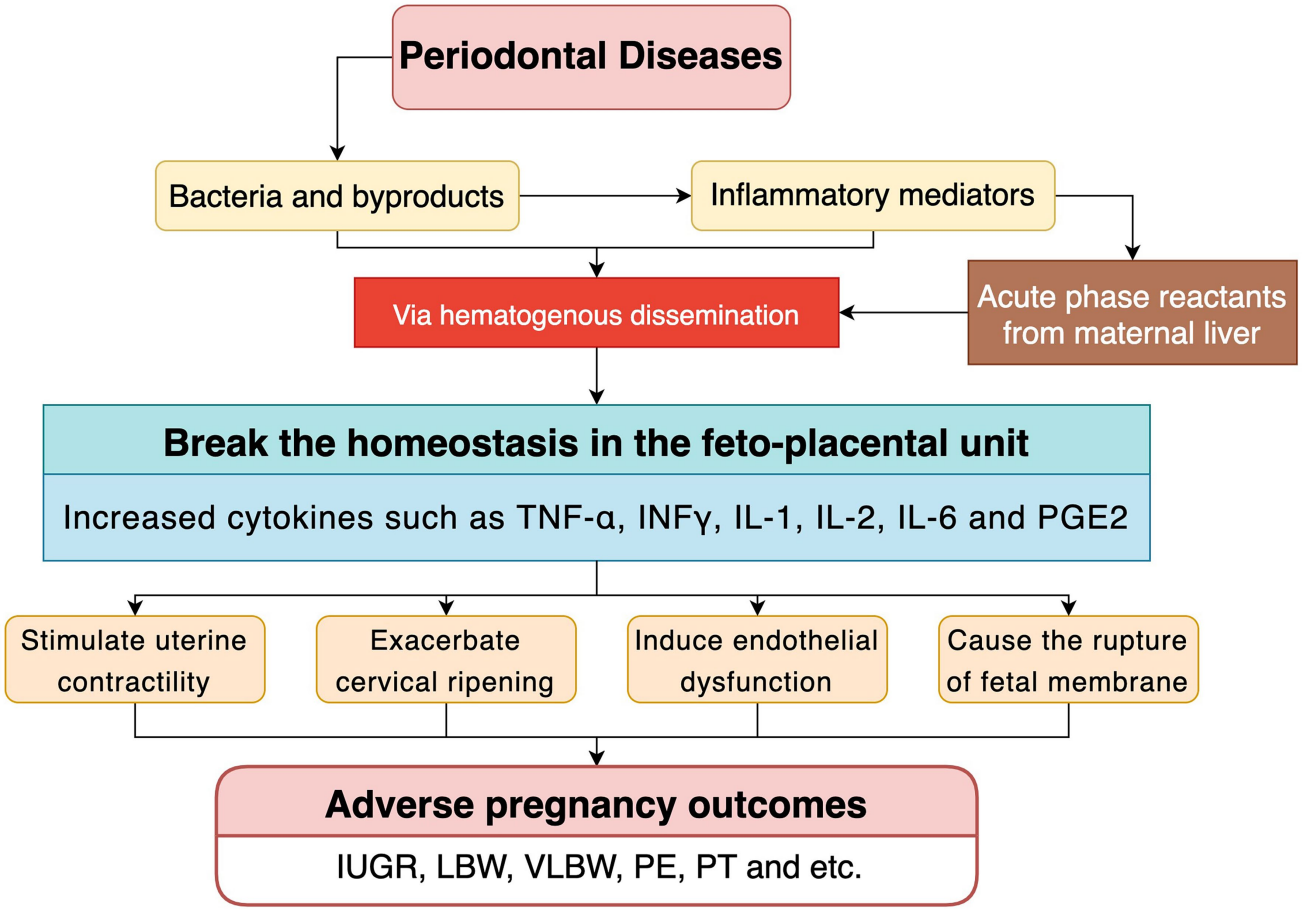
Inflammatory pathway and its role in adverse pregnancy outcomes.

Specifically, the enhanced inflammatory cytokine levels in the feto-placental unit stimulate uterine contractility, exacerbate cervical ripening, induce endothelial dysfunction, cause the rupture of fetal membrane and eventually leads to an increased risk for IUGR, LBW, VLBW, PE, PT and so on (Bobetsis et al., 2020; Gómez et al., 2020). Figure 2 presents the inflammatory pathway and its role in adverse pregnancy outcomes.

### 2.3. Direct infection: Microorganisms pass placenta and cause infection within fetal-placental unit

The fetal placental unit may not be as sterile as previously thought, with nearly a third of placental specimens confirmed to contain intracellular bacteria in the substrate, the tissue layer and the underside of the maternal-fetus interface (Pelzer et al., 2017; Park et al., 2022). Moreover, a placenta microbiome study reported that the placental microbiome is in fact more closely related to the oral microbiome than to the urogenital tract microbiome, which is what is generally expected. This finding suggested a link between oral microbiome and potential adverse pregnancy outcomes (Aagaard et al., 2014; Zi et al., 2014).

The infected periodontal tissues acts as a reservoir for bacteria that can transfer from periodontal tissues to the fetal placenta unit and trigger a metastatic infection (Zi et al., 2014; Bobetsis et al., 2020; Genco and Sanz, 2020). Periodontal pathogens, such as *Fusobacterium nucleatum*, *Porphyromonas gingivalis*, *Filifactor alocis*, *Campylobacter rectus*, *Tannerella forsythia*, *Prevotella nigrescens*, and *Parvimonas micra*, among others, were detected in the amniotic fluid from mothers with periodontal disease (Han et al., 2006; Andonova and Iliev, 2021; Narita and Kodama, 2022).

How do the oral microorganisms with their by-products actually contribute to adverse pregnancy outcomes? The pathogenic subgingival microorganisms make the translocation to the bloodstream possible, that is, they cause bacteremia (Figuero et al., 2020). The dissemination of symbiotic and pathogenic microbes in the blood can lead to the establishment of metastatic infections in various parts of the fetal-placental unit, such as amniotic fluid, choriodecidual space, placenta, chorioamniotic membrane, umbilical cord, and the fetus (Bobetsis et al., 2020).

A unique adhesin, namely Fad A, plays an important role in *F. nucleatum* (a Gram-negative anaerobe frequently associated with adverse pregnancy outcomes) colonization *in vivo* (Liu et al., 2007; Han et al., 2010). It is not only an adhesin but also an invasin (Han, 2015). Vascular endothelial-cadherin, a member of the cadherin family and a cell–cell connection protein, has been recognized as an endothelium receptor for Fad A, a necessary component for *F. nucleatum* to bind to endothelial cells. Due to the enhanced endothelial permeability, the bacteria are able to pass through loosening junctions in the endothelium and penetrate the placental barrier (Fardini et al., 2011).

Lipopolysaccharide (LPS), synthesized by pathogenic microorganisms, is one of the most important virulence factors. *Porphyromonas gingivalis* is the main pathogen of periodontal disease (Gómez et al., 2020; Mei et al., 2020). *Porphyromonas gingivalis* LPS induces IL-8 and IL-6 production *via* TLR-2 in chorion-derived cells and can increase expression of COX-2, IL-8 and TNF-α in human trophoblast-8 in an NF-κB-dependent fashion (Hasegawa-Nakamura et al., 2011; Ao et al., 2015). *Aggregatibacter actinomycetemcomitans* LPS (Aa-LPS) induces apoptosis in human trophoblasts *via* the mitochondria-dependent pathway by increasing levels of caspase 9, caspase 3, caspase 2, cytochrome c and so on (Li et al., 2011).

Moreover, there are many different molecular mechanisms specific to different microorganisms. For example, *P. gingivalis* can induce a decrease of CD56^+^ dNK cells and a rise in CD16^+^ dNK cells in the first trimester. It may also interrupt the function of stromal cells that are frequently linked to uNK cells and CD68^+^ macrophages in a paracrine manner (Reyes et al., 2017). Inadequate remodeling of the myometrial segments of the uterine spiral arteries, known as defective deep placentation (DDP), may also be a common mechanism of *P. gingivalis* inducing adverse pregnancy outcomes (Brosens et al., 2011). And in a dose-dependent manner, *Campylobacter rectus* challenge dramatically increased both mRNA and protein levels of TNF-α and IL-6 in human trophoblasts (Arce et al., 2010).

Lastly, circulating microorganisms together with their by-products may also trigger a direct inflammatory reaction in the uterus, which have been covered in detail in the indirect infection section (Zi et al., 2014; Bobetsis et al., 2020; Genco and Sanz, 2020).

### 2.4. Impact of immune system in pregnancy against periodontal pathogens leads to a secondary indirect attack on the fetal-placental unit

The infection induced by the microbial community in subgingival sites of periodontal disease patients leads to a maternal immune response to pathogenic bacteria and their products, and the elevated serum inflammatory cytokines produced by the immune system play an adverse role to the pregnancy. Nonetheless, this side-effect is listed as the third mechanism in this review, in addition to the indirect and direct effects.

Maternal immune responses play a dual role throughout pregnancy. On the one hand, the mother and her fetus must be shielded from external pathogens. On the other hand, the embryo/fetus expresses paternal antigens that serve as an allograft, which have to be tolerated by the mother during the whole pregnancy period (Zi et al., 2014; Bobetsis et al., 2020). Altogether, pregnancy characteristically presents with physiological immune tolerance.

Even in the early stages of pregnancy, the maternal immune system experiences significant changes. Specifically, some substances contained in seminal fluid can promote the shift of dendritic cells (DCs) to be more tolerogenic. This promotes a conversion from Th17 and T helper-1 (Th1) toward a T regulatory cells (Treg) and Th2. Treg may be involved in inhibiting maternal effector T cells, such as Th17 cells, in peripheral blood. In addition, antibodies secreted by B cells protect the presence of paternal antigens in trophoblasts once they enter the fetal-maternal interface. Last but not least, a wide range of molecules play a role in immune tolerance at the interface. For example, molecules secreted or produced by the trophoblast itself modulate the phenotype of function of immune cells, which can make DCs turn or remain immature and thus tolerogenic. When it comes to molecules secreted by innate immune system cells, they can positively influence trophoblast physiology while helping maternal T cells become or remain resistant to paternal antigens expressed by the fetus (Zenclussen, 2013; Bobetsis et al., 2020).

These physiological processes are so delicate that if any triggering mechanisms disturb them may break the balance and result in adverse pregnancy outcomes. Unfortunately, the infection of periodontal microbes triggers a switch in the maternal immune response to a pathogenic pro-inflammatory response, disrupting the homeostasis at the maternal-fetal interface and diminishing these immunological privileges throughout pregnancy (Zi et al., 2014).

Certain infectious diseases, even subclinical infections, may lead to an overall bias toward type 1, resulting in an increase in the number and activity of Th1/Th17 cells. The Th1 response activates decidual macrophages, which release toxic amounts of TNF-α and nitric oxide, leading to deleterious effects to the fetus. Overall, it appears that an imbalance of Th17/Treg proportion is associated with adverse pregnancy outcomes (Zenclussen, 2013; Zi et al., 2014).

The B cell response induced by infection cannot be neglected. Periodontal infection by *P. gingivalis* can cause atopobiosis to the placenta and induce inflammation (Gómez et al., 2020). Generally, the infection levels of *P. gingivalis* are correlated with the antibody response to the pathogens (Saraiva et al., 2014). A study has shown that LBW was linked to a higher maternal serum antibody level against *P. gingivalis* at mid-trimester (Dasanayake et al., 2001). Interestingly, in women with severe periodontitis, the risk of adverse pregnancy outcomes was higher when the antibody response to periodontal pathogens is lower (Zi et al., 2014).

### 2.5. Clinical interventions

Although a great deal of prospective studies have shown a positive association between oral condition and adverse pregnancy, the findings are far from conclusive when it comes to clinical interventions. There are still some controversial studies reporting that treating periodontal disease does not reduce the incidence of adverse pregnancy outcomes (Macones et al., 2010; Polyzos et al., 2010). Michalowicz et al. (2009) stated that non-surgical mechanical periodontal treatment did not significantly alter the level of inflammatory mediators in serum and these markers were not associated with adverse pregnancy outcomes such as PT and LBW. A study performed by Penova-Veselinovic et al. (2015) showed periodontal treatment can lower the levels of specific inflammatory mediators in gingival crevicular fluid, but no discernible difference in pregnancy outcomes was observed between the treatment and control groups. Reddy et al. conducted a randomized clinical study to establish the effect of non-surgical periodontal therapy on pregnancy outcomes in women with periodontal diseases. Phase-I periodontal therapy was given to the treatment group, while only oral hygiene guidance was imparted to the control group. The results showed no statistically significant difference in pregnancy outcomes between the groups. However, this study concluded that periodontal diseases enhanced the serum IgM antibody concentration, which may lead to a higher prevalence of PT and LBW in the control group (Reddy et al., 2014). Additionally, a multivariate logistic analysis performed by López et al. (2005) showed that women with periodontal diseases were at a higher incidence of PT/LBW than women who received periodontal therapy before 28 weeks of gestation.

As in previous literature, recent studies have demonstrated that periodontal diseases are risk factors for adverse pregnancy outcomes. However, the impact of periodontal treatment on the prevention of adverse pregnancy outcomes remains a contentious topic, despite the fact that the majority of non-surgical mechanical treatments for pregnant women with periodontal disease have shown improvements in clinical parameters regarding oral health. Given the complexity of the clinical problem, the inconsistent data could be attributed to variations in periodontal disease diagnosis criteria, the effectiveness of periodontal treatment strategies, individual differences in maternal responses, differences in disease severity, and so on. Notably, significant associations have been reported with antenatal factors and periodontal status leading to adverse pregnancy outcomes. For example, maternal stress is a risk factor for PT, which may be related to the production of adrenocorticotropic hormone-releasing hormone (CRH; Mannem and Chava, 2011; Romero et al., 2014). Other relative heterogeneities in the study population, such as smoking, age, ethnicity, and education level may also influence pregnancy outcomes (Huck et al., 2011).

How can the occurrence of adverse pregnancy outcomes be minimized? First, periodontal treatment before pregnancy is recommended. The first 12 weeks of pregnancy are crucial for fetal organogenesis and therefore aggressive periodontal treatment is not recommended during this period. From a biological point of view, treatment given in the second trimester may be too late because the pathogenicity potential of the microbial community and the severity of the periodontal disease increase throughout the pregnancy and interventions at this stage cannot influence pathogens already present in the placenta (Zi et al., 2014; Cobb et al., 2017). Next, surgical treatment and the use of antibiotics can be taken into account. More aggressive treatments such as surgical periodontal treatment can better improve periodontal conditions, especially for patients with severe periodontal disease (Heitz-Mayfield et al., 2002).The additional application of antibiotics including amoxicillin and metronidazole may serve as an effective intervention to get rid of periodontal diseases (Leitich et al., 2003; Keestra et al., 2015). However, given the specificity of the pregnant population, the fear of teratogenicity may mean that there is still a long way to go before these can be implemented. While undergoing more thorough treatment, dentists prescribing medication to pregnant women should specifically follow the Food and Drug Administration (FDA) regulations regarding the use of medication in pregnancy. Lastly, there should be interdisciplinary cooperation between obstetricians and dentists in order to efficiently identify risk factors for adverse pregnancies and to provide timely and effective interventions. Preventive oral health care can also be promoted as part of prenatal care. Obstetricians, as the health care professionals most commonly contacted by women during pregnancy, should educate on the importance of maintaining good oral hygiene and promptly remind them to receive necessary dental care.

## 3. Pregnancy alters the progression of periodontal diseases

Periodontal health impacts the pregnancy process, and vice versa. In other words, not only may periodontal disease interfere with pregnancy but also periodontal conditions tend to worsen during pregnancy due to specific physiological alterations (Kapila, 2021).

During pregnancy, a woman’s body goes through significant hormonal changes and organ system adaptations, as well as changes in the oral cavity. Hormones, as specific regulatory molecules, play important roles in modulating the periodontal tissue responses and may change periodontal tissue responses to microbial plaque, which could exacerbate the severity of periodontal disease (Güncü et al., 2005; Yokoyama et al., 2005). During pregnancy, a woman’s sex hormones levels fluctuate wildly. Progesterone and estrogen, which work through various biochemical mechanisms to quiet or activate the myometrial smooth muscle cervical composition and reach peak plasma levels by the end of the third trimester, mediate the majority of the hormonal regulation of labor and birth (Bobetsis et al., 2020; Figuero et al., 2020). These hormone changes make the host more susceptible to periodontal disease.

### 3.1. Increased inflammatory response

Increased sensitivity to stimuli occurs in the gingiva during pregnancy (Terzic et al., 2021). For example, pregnant women are more susceptible to inflammation and symptoms often take place in the second or third month of pregnancy. When probed, the gingiva seem red, swollen, sensitive to stimulation, larger, and prone to bleeding (Huck et al., 2011; Gare et al., 2021).

Sex hormones can modulate the production of cytokines. The temporary elevation of certain sex hormones such as progesterone and estrogen can induce proinflammatory cytokines including IL-6, IL-8, and IL-1β to be released in the tissue, which has been associated with an increase in the extent, prevalence and intensity of gingival inflammation (Bobetsis et al., 2020; Figuero et al., 2020). Moreover, progesterone increases the synthesis of prostaglandins, particularly PGE2, which can amplify the clinical manifestations of gingival inflammation by increasing vascular capillarity and permeability (Markou et al., 2009).

In addition to the vascular system, connective tissue is also a major target of hormones during pregnancy. The migratory cells, fibroblasts, and extracellular matrix can also be affected (Laine, 2002). Progesterone plays an important role in increasing the production of vascular endothelial growth factor (VEGF) in human gingival fibroblasts (HGF; Yokoyama et al., 2005). And it can dilate the gingival capillaries and increase capillary permeability by stimulating the endothelial cells through inhibiting cellular antioxidant effect and increasing oxidative stress (Prakash et al., 2012; Yuan et al., 2016). The changes in vascular responses and connective tissue turnover in the periodontium indirectly contribute to the increased gingival inflammation (Silva de Araujo Figueiredo et al., 2017).

### 3.2. Shifts on the composition of oral microorganisms

During pregnancy, the surge of hormonal levels triggers oral tissue responses (Cornejo Ulloa et al., 2021), which means changes in the composition or abundance of oral microorganisms occur relative to postpartum or non-pregnant status. This shift may lead to a potentially more hazardous microbial community (Zi et al., 2014).

Pregnancy, especially in the early stages, accelerates the growth of bacteria in the oral cavity and makes it easier for periodontal pathogens to colonize there (Fujiwara et al., 2017). A major change in the oral microbiome during pregnancy is increased microbial load (Neuman and Koren, 2017). Research examining the prevalence of seven common bacterial species in the oral cavity found that early pregnancy had considerably higher overall cultivable microbial counts compared to non-pregnant women. It is worth noting that the bacteria count of *P. gingivalis* and *A. actinomycetemcomitans*, two of the main periodontal pathogens, is elevated in pregnant women as well (Fujiwara et al., 2017). Progesterone levels in the first trimester are positively correlated with *P. gingivalis*, and this relevance suggests that progesterone levels during this period promote the growth of *P. gingivalis* (Massoni et al., 2019). This phenomenon can be explained by the fact that both estradiol and progesterone could substitute vitamin K, which serves as an essential growth factor for *P. gingivalis*, and therefore stimulates *P. gingivalis* growth and elevates gingival inflammation (Kornman and Loesche, 1982). It is also consistent with the fact that both progesterone and estradiol are significantly elevated during pregnancy. Moreover, increased levels of anaerobic species such as *A. actinomycetemcomitans* and *Parvimonas micra* may also induce a shift in the microbial communities on mucosal surfaces, which can lead to pro-inflammatory immune responses (Zi et al., 2014).

### 3.3. Suppression of the immune system

The maturation and selection of thymocytes, cell proliferation, MHC-II expression, cell migration and cytokine generation are all immunological processes that are modulated by sex hormones (Ortiz-Sánchez et al., 2021). The severity of periodontal diseases can be exacerbated by immune suppression during pregnancy, including the altered lymphocyte response, suppression of T-cell activity, decreased antibody production and depressed phagocytosis and neutrophil chemotaxis (Boyapati et al., 2021).

Specifically, during pregnancy, the immune system is adapted to be able to tolerate the fetus, a potential antigen source. Thus, both in the fetal-maternal interface and the peripheral blood, an immune response shift from Th1 and Th17 to Th2 and Treg cells takes place (Zi et al., 2014; Bobetsis et al., 2020). Also, functional changes in polymorphonuclear leukocytes include alterations and decreases of chemotaxis, as well as adherence and inhibition of the neutrophil respiratory burst, which can worsen the periodontal condition (Morelli et al., 2018). Additionally, proinflammatory cytokines such as IFN-γ and TNF-α may also decrease with the increase of estrogen, which has been observed in experimental models (Soldan et al., 2003). These modifications affect the defensive system of periodontal tissues, making gingival tissue less efficient at resisting the inflammatory challenges produced by bacteria (Gare et al., 2021; Raju and Berens, 2021).

The mother’s immune system is more vulnerable during this special period, making her body more susceptible to illnesses. It has been shown that human gingiva is a target tissue for increases in estrogen and progesterone. Moreover, periodontal microvascularization can be caused by estradiol. These changes in oral tissues lead to a transition toward a more anaerobic flora (Pucci et al., 2021), which favors the growth of periodontal pathogens.

### 3.4. Changes in mood and lifestyle habits

Emotional and psychosocial stress are factors of periodontal disease. The emotional fluctuation during pregnancy could increase the mother’s risk for periodontal disease, but the precise role of stress in the pathogenesis of periodontal diseases is unknown (Pihlstrom et al., 2005). Moreover, some women may modify their dietary habits during the first trimester of pregnancy, such as consuming more carbohydrates. And vomiting during this period increases the acidity of saliva (Morelli et al., 2018). As mentioned above, pregnancy aggravates gingiva bleeds due to the elevated concentration of estrogens, and the bleeding may make women feel unwilling to brush their teeth because of hemophobia (Terzic et al., 2021).

## 4. Conclusion

This review discusses the bidirectional relationship between periodontal disease and adverse pregnancy outcomes and elucidates the potential mechanisms. We further explored the underlying logic behind this bidirectional relationship from three possible pathways building on existing research.

Although current mechanistic and clinical intervention studies need to be further developed, clarification of the relationship between specific periodontal pathogens, inflammatory factors and adverse pregnancy outcomes can help to develop effective preventive intervention strategies for specific populations. Ultimately, periodontal disease is relatively both preventable and treatable, whereas adverse pregnancy outcomes can be a huge burden to the family and society. Therefore, prenatal periodontal treatment is a decent option because it improves oral health, advances general health, and reduces the risk of deleterious effects to the pregnant women and their fetuses.

## Author contributions

XW and XF contributed to the conception and design of the work, drafting the manuscript, made final approval of the version to be published, and agree to be accountable for all aspects of the work in ensuring that questions related to the accuracy or integrity of any part of the work are appropriately investigated and resolved. LY and CZ contributed to the interpretation of data for the work, made the figures, drafting the manuscript, made final approval of the version to be published, and agree to be accountable for all aspects of the work in ensuring that questions related to the accuracy or integrity of any part of the work are appropriately investigated and resolved. RH contributed to the conception and design of the work, revised the manuscript, made final approval of the version to be published, and agree to be accountable for all aspects of the work in ensuring that questions related to the accuracy or integrity of any part of the work are appropriately investigated and resolved. All authors contributed to the article and approved the submitted version.

## Funding

This study is partially supported by National Natural Science Foundation of China (NSFC31800114) to RH.

## Conflict of interest

The authors declare that the research was conducted in the absence of any commercial or financial relationships that could be construed as a potential conflict of interest.

## Publisher’s note

All claims expressed in this article are solely those of the authors and do not necessarily represent those of their affiliated organizations, or those of the publisher, the editors and the reviewers. Any product that may be evaluated in this article, or claim that may be made by its manufacturer, is not guaranteed or endorsed by the publisher.
